# Development of a novel genetic sexing strain of *Ceratitis capitata* based on an X-autosome translocation

**DOI:** 10.1038/s41598-023-43164-0

**Published:** 2023-09-27

**Authors:** Carlos Cáceres, Kostas Bourtzis, Georgia Gouvi, Marc J. B. Vreysen, Nanwintoum Séverin Bimbilé Somda, Martina Hejníčková, František Marec, José S. Meza

**Affiliations:** 1 Insect Pest Control Laboratory, Joint FAO/IAEA Centre of Nuclear Techniques in Food and Agriculture, International Atomic Energy Agency, 2444 Seibersdorf, Austria; 2 Unité de Formation et de Recherche en Sciences et Technologies (UFR/ST), Université Norbert ZONGO (UNZ), BP 376 Koudougou, Burkina Faso; 3 Biology Centre CAS, Institute of Entomology, 370 05 České Budějovice, Czech Republic; 4 Faculty of Science, University of South Bohemia, 370 05 České Budějovice, Czech Republic; 5 Programa Operativo de Moscas, SADER-SENASICA/IICA, Camino a los Cacaotales S/N, CP 30860 Metapa de Domínguez, Chiapas México; 6 Present Address: Department of Life Sciences, Imperial College London, Sir Alexander Fleming Building, South Kensington Campus, London, UK

**Keywords:** Biological techniques, Genetics, Zoology

## Abstract

Genetic sexing strains (GSS), such as the *Ceratitis* *capitata* (medfly) VIENNA 8 strain, facilitate male-only releases and improve the efficiency and cost-effectiveness of sterile insect technique (SIT) applications. Laboratory domestication may reduce their genetic diversity and mating behaviour and hence, refreshment with wild genetic material is frequently needed. As wild males do not carry the T(Y;A) translocation, and wild females do not easily conform to artificial oviposition, the genetic refreshment of this GSS is a challenging and time-consuming process. In the present study, we report the development of a novel medfly GSS, which is based on a viable homozygous T(XX;AA) translocation using the same selectable markers, the *white* *pupae* and *temperature-sensitive* *lethal* genes. This allows the *en* *masse* cross of T(XX;AA) females with wild males, and the backcrossing of F_1_ males with the T(XX;AA) females thus facilitating the re-establishment of the GSS as well as its genetic refreshment. The rearing efficiency and mating competitiveness of the novel GSS are similar to those of the T(Y;A)-based VIENNA 8 GSS. However, its advantage to easily allow the genetic refreshment is of great importance as it can ensure the mass production of high-quality males and enhanced efficacy of operational SIT programs.

## Introduction

Tephritid species, such as *Anastrepha* *ludens,* *Bactrocera* *dorsalis*, *Ceratitis* *capitata* and *Zeugodactus* *cucurbitae*, are among the most important fruit and vegetable pests in the world, being responsible for significant crop losses on all continents^1^. The sterile insect technique (SIT) has been used for the suppression, containment, prevention of (re)establishment or local eradication of populations of these tephritid pests^2,3^ The SIT is based on the mass-rearing, sterilization using ionizing irradiation, handling, transport, and release of sterile insects, ideally only males, over the target area where sterile males will compete with wild males for mating with wild females^4^. Through sequential releases of sterile males to obtain adequate overflooding ratios, the target population control goal (suppression, containment, prevention, or local eradication) will be achieved^5,6^.

Although fruit fly SIT programs have been successfully carried out by releasing both sterile males and females, it has been shown that the release of only sterile males can significantly enhance the efficiency and cost-effectiveness of the application^7–11^. Sterile male-only releases for tephritid species have been achieved through the development of genetic sexing strains (GSS) using as a selectable morphological marker, e.g. the colour of the puparium^12–20^. In these GSS, male pupae have the wild-type brown colour while females are mutant with white or a black pupal colour. These phenotypes are determined by recessive alleles of single copy genes^8,21–23^. The different colouration of male and female pupae is achieved by linking the wild-type allele of the respective pupal colour genes to the male determining region via a Y-autosome reciprocal translocation T(Y;A)^8,12,21,22^. Such translocation lines and their respective GSS are semi-sterile since only half of the gametes, those produced via alternate segregation, are genetically balanced, viable and fertile^8,24^. White pupae-based GSS have been developed for *Ceratitis* *capitata*, *Bactrocera* *carambolae*, *B.* *dorsalis,* and *Z.* *cucurbitae*^13,15–17,20^. Black pupae-based GSS have been developed for *Lucilla* *cuprina,* *C.* *capitata,* *Anastrepha* *fraterculus* and *A.* *ludens*^12,13,18,19^. In all these GSS, the separation of males from females can be done at the pupal stage in support SIT applications against these species.

The GSS of *C.* *capitata* and *A.* *ludens*, are being used in large scale operational SIT programs^25,26^. A second generation GSS of *C.* *capitata* carries, in addition to the *white* *pupae* (*wp)* marker, a *temperature* *sensitive* *lethal* (*tsl*) gene^27^. The *wp* and *tsl* genes are localized on chromosome 5 and are closely linked. Thus, in a reciprocal translocation T(Y;5) involving the *wp* *tsl* genomic region, the wild-type alleles of *wp* and *tsl* genes are linked to *Maleness-on-the-Y* (*MoY*), the male determining gene^28^.Therefore, males emerge from brown pupae and are resistant to elevated temperatures (34–35 °C) while females emerge from white pupae and are sensitive to this temperature, e.g. the female embryos will die when exposed to these temperatures for 24 h^8,22^.

Although the development of T(Y;A) reciprocal translocations using irradiation is relatively easy, the GSS based on these translocations face some challenges such as recombination which may affect the genetic stability of the strain resulting in the production of wild-type females and mutant males^29^. These recombination events can be controlled by implementing a filter rearing system (FRS) that allows the removal of recombinants^30^. The incorporation of a chromosomal inversion covering the genomic region of interest can also be used as a biological filter since inversions are known recombination suppressors^8,31^.

Laboratory domestication is another major challenge to maintain the quality, including the mating behavior of the GSS, be it under small-scale or mass-rearing conditions. Reduced competitiveness of the released sterile males will have detrimental effects on the efficiency of the SIT^32^. In such cases, the usual process is a genetic refreshment of the laboratory colony with insects collected in nature. However, as wild males and wild females do not carry the translocation and the recessive alleles of the selectable markers used for the development of the GSS, respectively, it is impossible to implement a crossing scheme *en* *masse* that would allow the transfer of wild genetic material to refresh the laboratory colony of the GSS.

**Figure 1:**
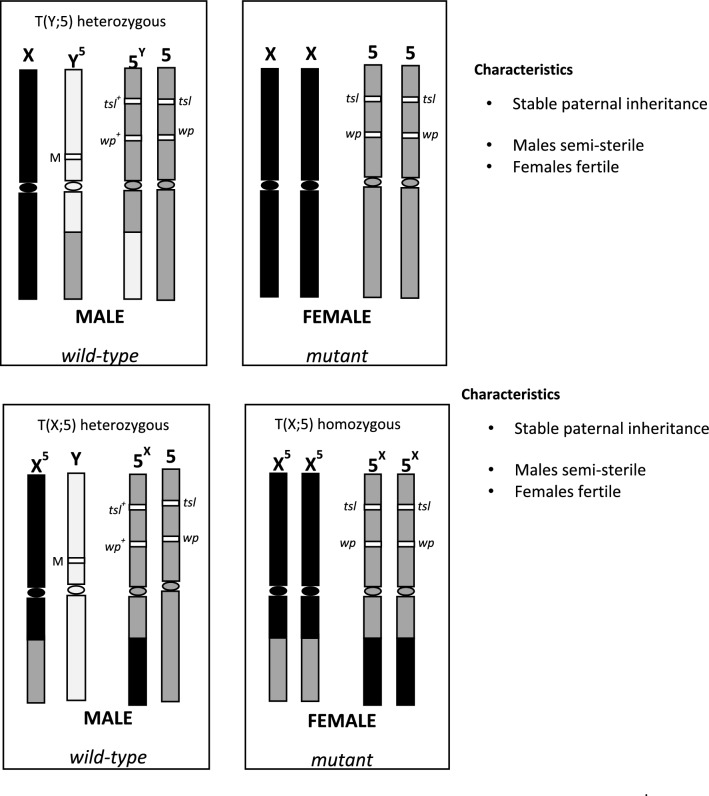
Structure and characteristics of the *C.* *capitata* T(Y;A) and T(XX;AA) genetic sexing strains.

This study presents a novel GSS that would enable refreshing the laboratory or mass-reared colonies through the *en* *masse* crossing of colony females with wild males. There are two important requirements for this new system: (a) females must carry the selectable marker(s) translocated to the X chromosome and (b) the T(X;A) translocation should be viable in homozygous condition [T(XX;AA)]. So, males will carry a free Y chromosome and a free autosome that will be carrying the dominant allele(s) of the selectable marker(s) and one copy of the chromosomes involved in the reciprocal translocation (Fig. 1). Using the selectable markers *wp* and *tsl*, we report the development and comparative evaluation of this novel *C.* *capitata* GSS, which is based on a T(X;A) homozygous translocation, with the T(Y;A)-based VIENNA 8 GSS currently used in mass-rearing facilities and large-scale operational programs worldwide.

## Results

### Development of novel *Ceratitis capitata* GSS based on homozygous T(X;5) translocations

**Figure 2:**
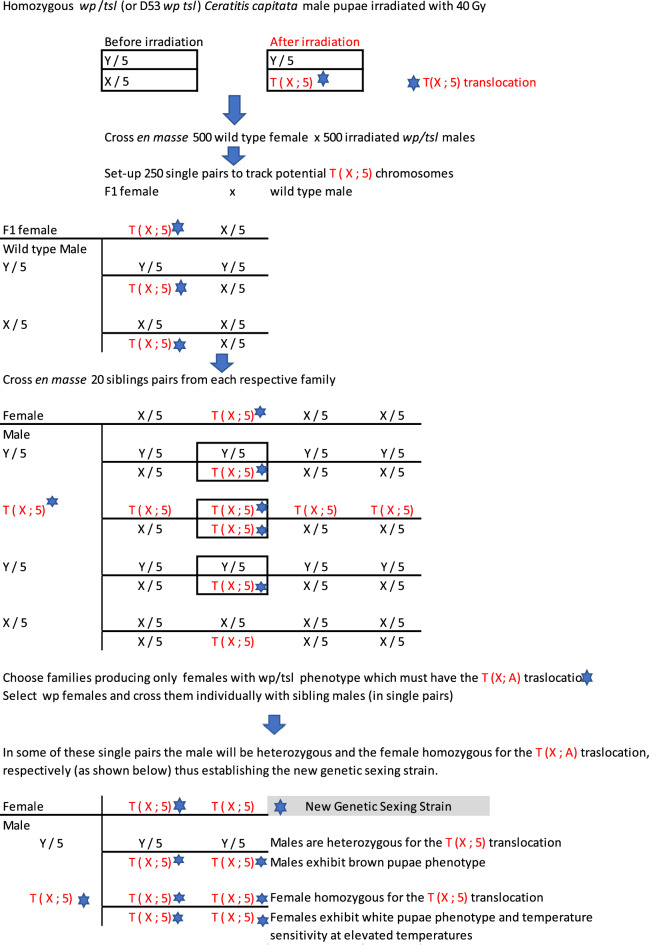
Schematic depiction of the protocol followed for the development of the homozygous *Ceratitis* *capitata* T(X;5) GSS.

The experimental procedure which was used for the development of novel *C*. *capitata* GSS’s based on homozygous T(X;5) translocations is presented in Fig. 2. Three GSS were constructed, one with the inversion D53 (Cc TX IPCL-2^D53^) and two without (Cc TX IPCL-1^D53-^ and Cc TX IPCL-3^D53-^). For the GSS without the inversion, the males used for the initial irradiation step for the induction of the translocation originated from the homozygous mutant line *wp* *tsl*. For the GSS with the inversion, the males used for irradiation were derived from the inversion line D53 *wp* *tsl*, which is homozygous for the inversion D53 and the mutant alleles of the *wp* and *tsl* genetic loci.

### Characterization of the novel T(X;5)-based GSS

All data presented below refer to the comparative characterization of two novel GSS (Cc X IPCL-1^D53-^ and Cc TX IPCL-2^D53^) with the VIENNA 8^D53-^ and are summarized in Table 1.

**Table 1:** Comparative characterization of the two newly developed medfly GSS, Cc TX IPCL-1^D53-^ and Cc TX IPCL-2^D53^, with the VIENNA 8^D53-^ GSS.

Production parameters^#^	VIENNA 8^D53-^	Cc TX IPCL-1^D53-^	Cc TX IPCL-2^D53^
	Average ± SE	Average ± SE	Average ± SE
Fecundity—egg/female/day during the first 12 days of oviposition	41.06a ± 1.79	34.48b ± 1.44	35.69b ± 1.59
Days average male larvae development time	10.09b ± 0.53	8.81a ± 0.21	8.34a ± 0.18
Days average female larvae development time	13.23c ± 0.05	9. ± 0.18	9.10a ± 0.07
Egg to pupae production rate (%)	75.57b ± 1.68	87.76a ± 2.58	88.81a ± 1.54
Egg to adult production rates (%)	69.20b ± 1.64	82.77a ± 2.48	81.17a ± 1.5
Female production rate (%)	31.20b ± 0.77	39.24a ± 0.84	39. ± 0.67
Male production rate (%)	37.91b ± 1.15	43.53a ± 1.69	41.40a ± 1.05
Male pupae production rate (%) from eggs treated at 35 °C	37.10b ± 1.03	41.08b ± 1.21	43.08a ± 1.8
Male adult production rate (%) from eggs treated at 35 °C	34.69b ± 1.04	38.04a ± 0.9	40.21a ± 1.59

Parameter values followed by the same letter are not statistically different.

#All production parameter values were determined at 24 °C unless otherwise is indicated (*p* < 0.05).

*Fecundity*: The VIENNA 8 ^D53-^ strain produced an average of 41.06 ± 1.79 eggs per female per day whereas the Cc TX IPCL-1^D53-^ and the Cc TX IPCL-2^D53^strains produced an average of 34.48 ± 1.59 and 35.71 ± 1.44 eggs per female per day, respectively. Statistical analysis indicated significant differences between the three strains (χ^*2*^ = 166.63, *p* < 0.001), with VIENNA 8^D53-^ clearly producing more eggs than both Cc TX IPCL-1^D53-^ (z ratio = 9.709, *p* < 0.001) and Cc TX IPCL-2^D53^ GSS (z ratio = 12.024, *p* < 0.001). The Cc TX IPCL-1^D53-^ and Cc TX IPCL-2^D53^ GSS produced statistically similar numbers of eggs per female per day (z ratio = − 2.327, *p* = 0.0521) (Supplementary file).

*Egg* *hatch*: The mean egg hatch for the VIENNA 8^D53-^, Cc TX IPCL-1^D53-^, and Cc TX IPCL-2^D53^ GSS, was statistically significantly different (χ^2^ = 86.79, *p* < 0.001), i.e. 87.61 ± 0.55%, 91.21 ± 1.38%, and 92.01 ± 0.94%, respectively. The eggs of the VIENNA 8^D53-^ strain showed a significantly lower hatch rate as compared with the hatch rate of eggs of the Cc TX IPCL-2^D53^ (z ratio = − 8.555, *p* < 0.001) and the Cc TX IPCL-1^D53-^ GSS (z ratio = − 6.901, *p *<  0.001). Hatch rate of eggs produced by the two novel T(X;A)-based GSS was similar (z ratio = − 1.709, *p* = 0.2018).

### Larval development time

Larval development time was statistically significant different between the strains (χ^2^ = 901.64, *p* < 0.001), sex (χ^2^ = 353.26, *p* < 0.001) and the interaction between strain and sex (χ^2^ = 165.48, *p* < 0.001) (Table 1).

Cc TX IPCL-1^D53-^ and Cc TX IPCL-2^D53^ GSS males completed their larval development significantly faster (8.81 ± 0.21 days and 8.34 ± 0.18 days, respectively) as compared with VIENNA 8^D53-^ males (10.09 ± 0.53 days) (t ratio = 8.03, df = 30, *p* < 0.001 and t ratio = 10.94, df = 30, *p* = 0.001, respectively). The development time of males was also significantly different between the Cc TX IPCL-1^D53-^ and Cc TX IPCL-2^D53^ strains (t ratio = 2.91, df = 30, *p* = 0.018).

**Figure 3:**
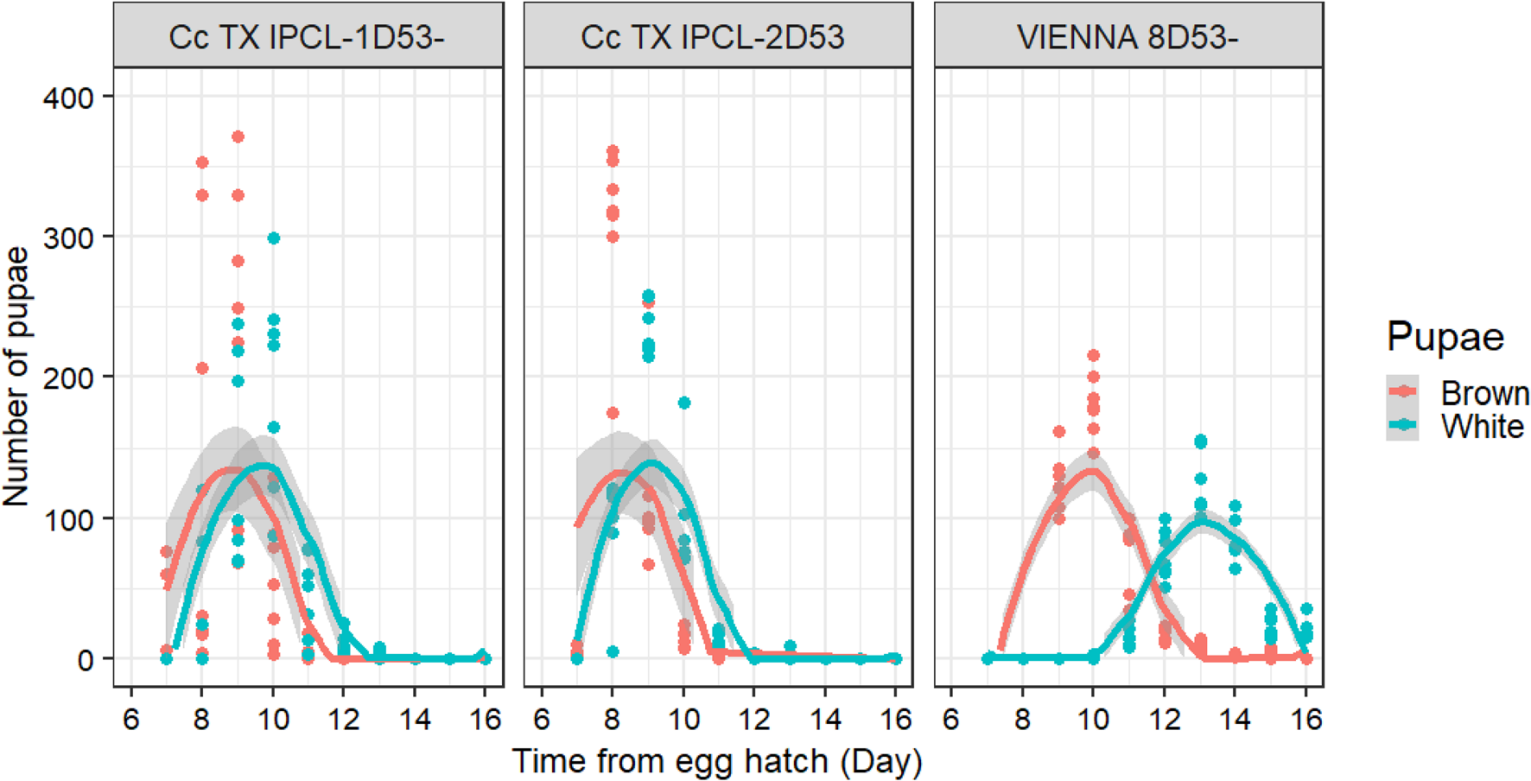
Pupation curve of three *Ceratits* *capitata* GSS: Cc TX IPCL-1^D53-^, Cc TX IPCL-2^D53^ and VIENNA 8^D53-^. The curves show the average number of larvae which reached pupation at a given day. The shadows surrounding the curves indicate the standard error with 95% confidence interval. The dots correspond to the number of pupae recorded per day. In the absence of recombination, males will emerge from brown pupae and females will emerge from white pupae.

Cc TX IPCL-1^D53-^ and Cc TX IPCL-2^D53^ females required significantly fewer days (9.71 ± 0.18 days and 9.10 ± 0.07 days, respectively) to complete their larval development as compared with VIENNA 8^D53-^ females (13.23 ± 0.05) (t ratio = 22.13, df = 30, *p* < 0.001 and t ratio = 25.974, df = 30, *p* < 0.001, respectively). Females of the Cc TX IPCL-2^D53^ strain completed their larval development significantly faster than females of the Cc TX IPCL-1^D53-^ strain (t ratio = 3,84, df = 30, *p* = 0.017). (Fig. 3).

### Egg to pupae production rate (%)

The mean pupal production rate (percentage of pupae as proportion of eggs) was significantly different between the three strains, i.e., 75.44 ± 1.68% for VIENNA 8^D53-^, 87.56 ± 2.58% Cc TX IPCL-1^D53-^ and 88.81 ± 1.54% for Cc TX IPCL-2^D53^ (χ^2^ = 555.79, *p* < 0.001). The VIENNA 8^D53-^ strain produced significantly fewer pupae as compared with the Cc TX IPCL-1^D53-^ and Cc TX IPCL-2^D53^ strains (z ratio = –18.53, *p* < 0.001 and z ratio = − 20.25, *p* < 0.001, respectively) while there were no differences between the two Cc TX strains (z ratio = − 1.94, *p* = 0.125) (Supplementary file).

### Egg to adult production rate or adult production rate by sex (%)

The mean adult production rate (percentage of adults as proportion of eggs) was significantly different between the three strains, i.e., 69.20 ± 1.64% for VIENNA 8^D53-^, 82.77. ± 2.48% for Cc TX IPCL-1^D53-^ and 81.17 ± 1.50% for Cc TX IPCL-2^D53^ (χ^2^ = 436.49, *p* < 0.001). The VIENNA 8^D53-^ strain produced significantly fewer adults than the Cc TX IPCL-1^D53-^ and Cc TX IPCL-2^D53^ strains (z ratio = − 18.61, *p* < 0.001 and z ratio = − 16.28, *p* < 0.001, respectively), while the adult production rate was also significantly different between the two Cc TX strains (z ratio = 2.46, *p* = 0.037) (Supplementary file).

Male and female adult production rates were significantly different between strains and within strains (F = 25.63, *p* < 0.001; F = 24.19, *p* < 0.001 respectively). The mean female production rate from eggs was 31.29 ± 0.77% for VIENNA 8^D53-^, 39.24 ± 0.84% for Cc TX IPCL-1^D53-^ and 39.77 ± 0.67% for Cc TX IPCL-2^D53^. The VIENNA 8^D53-^ strain produced significantly fewer females than the Cc TX IPCL-1^D53-^ and Cc TX IPCL-2^D53^ strains (z ratio = − 6.087, *p* < 0.001 and z ratio = − 6.458, *p* < 0.001, respectively), whereas there was no difference in female production rate between the two Cc TX strains (z ratio = − 0.373, *p* = 0.93). The mean male production rate from eggs was 37.91 ± 1.15% for VIENNA 8^D53-^, 43.53 ± 1.69% for Cc TX IPCL-1^D53-^ and 41.40 ± 1.05% for Cc TX IPCL-2^D53^. The male production rate of the VIENNA 8^D53-^ strain was significantly lower than that of Cc TX IPCL-1^D53-^ (z ratio = − 3.874, *p* < 0.001), but similar to that of the Cc TX IPCL-2^D53^ strain (z ratio = − 2.45, *p* < 0.038). No significant difference was found between the male production rate of the Cc TX IPCL-1^D53-^ and Cc TX IPCL-2^D53^ strains (z ratio = − 1.42, *p* < 0.3282).

The Cc TX IPCL-2^D53^ strain produced similar numbers of females and males (z ratio = − 1.13, *p* = 0.26), whereas the VIENNA 8^D53-^and Cc TX IPCL-1^D53-^ strains produced significantly fewer females than males (z ratio = − 5.14, *p* < 0.001 and z ratio = − 2.92, *p* = 0.004, respectively).

### Temperature sensitivity

The mean egg hatch after the temperature treatment at 35 °C was 50.44 ± 1.10% for VIENNA 8^D53-^, 46.40 ± 0.69% for Cc TX IPCL-1^D53-^ and 46.42 ± 1.40% for Cc TX IPCL-2^D53^. Statistical analysis indicated that egg viability of the VIENNA 8^D53-^ strain was significantly higher as compared with both the Cc TX IPCL-1^D53-^ and Cc TX IPCL-2^D53^ strains (z ratio = − 4.78, *p* < 0.001 and z ratio = 4.75, *p* < 0.001, respectively). Egg viability between the two Cc TX strains was similar (z ratio = − 0.03, *p* = 0.99) (Supplementary file).

The mean male pupae production rate of the three strains was significantly different after the temperature treatment of the eggs at 35 °C (χ^2^ = 30.34, *p* < 0.001), i.e., 37.07 ± 1.03% for VIENNA 8^D53-^, 41.08 ± 1.21% for Cc TX IPCL-1^D53-^and 43.08 ± 1.80% for Cc TX IPCL-2^D53^. The VIENNA 8^D53-^ strain produced significantly fewer male pupae as compared with the Cc TX IPCL-1^D53-^ (z ratio = − 4.87, *p* < 0.001) and Cc TX IPCL-2^D53^ (z ratio = − 7.24, *p* < 0.001) strains, whereas the male production rate of the two Cc TX strains was similar (z ratio = − 2.38, *p* = 0.05) (Supplementary file).

The mean male adult production rate from eggs after the temperature treatment of the eggs at 35 °C was significantly different among the three strains (χ^2^ = 30.34, *p* < 0.001), i.e., 34.69 ± 1.04% for VIENNA 8^D53-^, 38.04 ± 0.93% for Cc TX IPCL-1^D53-^ and 40.21 ± 1.59% for Cc TX IPCL-2^D53^). Male adult production rate of the VIENNA 8^D53-^strain was significantly lower than that of the Cc TX IPCL-1^D53-^ and Cc TX IPCL-2^D53^ strains (z ratio = − 4.56, *p* < 0.001 and z ratio = − 6.75, *p* < 0.001, respectively) while the two Cc TX GSS did not show any difference (z ratio = − 2.19, *p* = 0.07) (Supplementary file).

### Male mating competitiveness

Male mating competitiveness was determined for the Cc TX IPCL-1^D53-^, Cc TX IPCL-2^D53^ and VIENNA 8^D53-^ strains under field cage conditions using two wild-type populations, i.e., Guatemala and Valencia. The overall mating success of the two wild-type populations, during the evaluation of the three GSS, was high (Guatemala 0.85 ± 0.06 and Valencia 0.67 ± 0.15), reflecting the good quality of the insects used and the appropriateness of the field cage environment. The males of all three GSS achieved significantly more matings than the Guatemala wild-type males: Guatemala (9.16 ± 0.49) versus Cc TX IPCL-1^D53-^ (13.0 ± 0.49) (F_1,10_ = 29.71, *p* < 0.01), Guatemala (9.0 ± 0.47) versus Cc TX IPCL-2^D53^ (12.6 ± 0.47) (F_1,10_ = 30.25, *p* < 0.01), Guatemala (8.5 ± 0.72) versus VIENNA 8^D53-^ (12.0 ± 0.72) (F_1,10_ = 11.66, *p* < 0.01). However no significant differences were observed using the Valencia wild-type males: Valencia (9.83 ± 0.66) versus Cc TX IPCL1^D53-^ (11.33 ± 0.66) (F_1,10_ = 2.57, *p* = 0.13), Valencia (9.5 ± 0.97) versus Cc TX IPCL-2^D53^ (9.00 ± 0.97) (F_1,10_ = 0.13, *p* = 0.72), Valencia (9.16 ± 0.99) versus Cc VIENNA 8^D53-^ (9.83 ± 0.99) (F_1,10_ = 0.22, *p* = 0.64). No significant difference was also detected in the RSI for the three GSS in comparison with the Guatemala population (F_2,15_ = 0.01, *p* = 0.98) and the Valencia population (F_2,15_ = 0.47, *p* = 0.63) (Table 1).

### Cytogenetics

**Figure 4:**
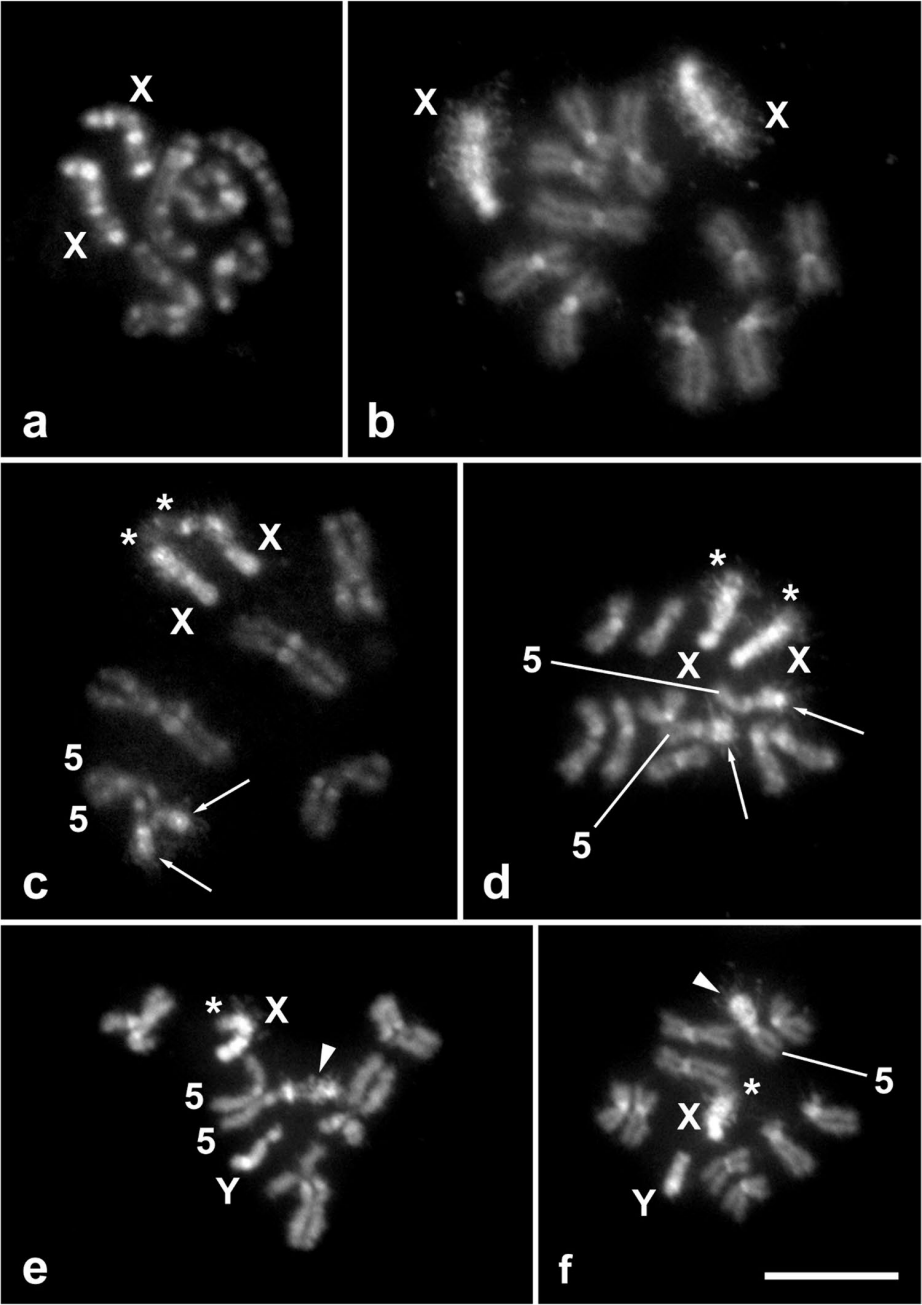
Cytogenetic analysis of the T(X;5) translocation strain of *Ceratitis* *capitata*. DAPI-stained mitotic chromosomes were obtained from the brains of wild-type larvae (**a**, **b**) and T(X;5) larvae (**c–f**). X and Y symbols denote sex chromosomes, symbol 5 stands for chromosome 5. Bar = 10 µm. (**a**) Female mitotic prometaphase with two X chromosomes and five pairs of somatically paired autosomes. (**b**) Female mitotic metaphase with two X chromosomes and five pairs of autosomes. (**c**) Female mitotic prometaphase showing reciprocal translocations between two X chromosomes (asterisks) and two somatically paired chromosomes 5 (arrows). (**d**) Female mitotic metaphase showing reciprocal translocations between two X chromosomes (asterisks) and two chromosomes 5 (arrows). (**e**) Male mitotic prometaphase showing a T(X;5) translocation on the X chromosome (asterisk) and a large T(5;X) translocation on one chromosome 5 (arrowhead). Note that the somatically paired homologue of chromosome 5 and the unpaired Y chromosome appear normal. (**f**) Male mitotic metaphase showing a T(X;5) translocation on the X chromosome (asterisk) and a large T(5;X) translocation on one chromosome 5 (arrowhead). Note that the homologue of chromosome 5 and the Y chromosome appear normal.

The diploid karyotype of *C.* *capitata* is 2n = 12, consists of five pairs of autosomes and one pair of sex chromosomes (XX females and XY males)^33^, and was confirmed in the wild-type strain used as a control in this study (Fig. 4a). In mitotic prometaphase of wild-type females, two X chromosomes were easily recognized by several large blocks of DAPI-positive heterochromatin, the number of which depended on the degree of chromosome condensation (see four blocks in Fig. 4a). The five autosome pairs were somatically paired at this stage, as is typical for flies^34,35^, and thus showed five instead of ten elements (Fig. 4a). In mitotic metaphase, two acrocentric X chromosomes were strongly highlighted with DAPI, indicating that they are largely composed of AT-rich heterochromatin (Fig. 4b). The smaller acrocentric Y chromosome in both the wild-type and the translocation Cc TX IPCL-3^D53-^ strain also consisted of DAPI-positive heterochromatin (Fig. 4e-f). In contrast, in the five pairs of submetacentric to acrocentric autosomes, both the short and long arms consisted of euchromatin, and only their centromeric regions had DAPI-positive heterochromatin blocks (Fig. 4b), similar to the C–bands in Canovai et al.^36^. Note that pairs of autosome homologues, although well separated by the spreading technique used, remained close to each other because of previous somatic pairing (Fig. 4b).

In females of the translocation strain, the terminal segments of the long, short heterochromatic arms of the X chromosomes were replaced by a segment of euchromatin, indicating that the X chromosomes carry a translocation from an autosome. This translocation was clearly visible in both the prometaphase and metaphase nuclei (see asterisks in Fig. 4c,d). From the experimental scheme (Fig. 2), it is clear that this translocation clearly originates from the short arm of submetacentric chromosome 5. Therefore, we confirm the interchromosomal rearrangement as a T(X;5) translocation. In addition, we observed a conspicuous heterochromatin block at the ends of the arms of an autosome pair in these females (see arrows in Fig. 4c,d). Most likely, this block of heterochromatin was translocated from the long arm of the X chromosome to the short arm of chromosome 5. Therefore, we interpret this interchromosomal rearrangement as a T(5;X) translocation. Taken together, we conclude that the Cc TX IPCL-3^D53-^ females are homozygous for a reciprocal translocation between X chromosome and short arms of chromosome 5.

The males of the translocation strain examined also carried a T(X;5) translocation on the single X chromosome, while the Y chromosome appeared normal, as in wild-type flies (Fig. 4e,f). However, the reciprocal T(5;X) translocation was only observed on one chromosome 5 (see arrowheads in Fig. 4e,f), while the other chromosome 5 was wild-type. This was particularly evident at the prometaphase stage, as the short arm of the intact chromosome 5 and the arm carrying the T(X;5) translocation differed greatly in length (Fig. 4e). The males of the translocation strain is thus heterozygous for the T(5;X) translocation.

### Localization of rDNA in wild-type and translocation strains

**Figure 5:**
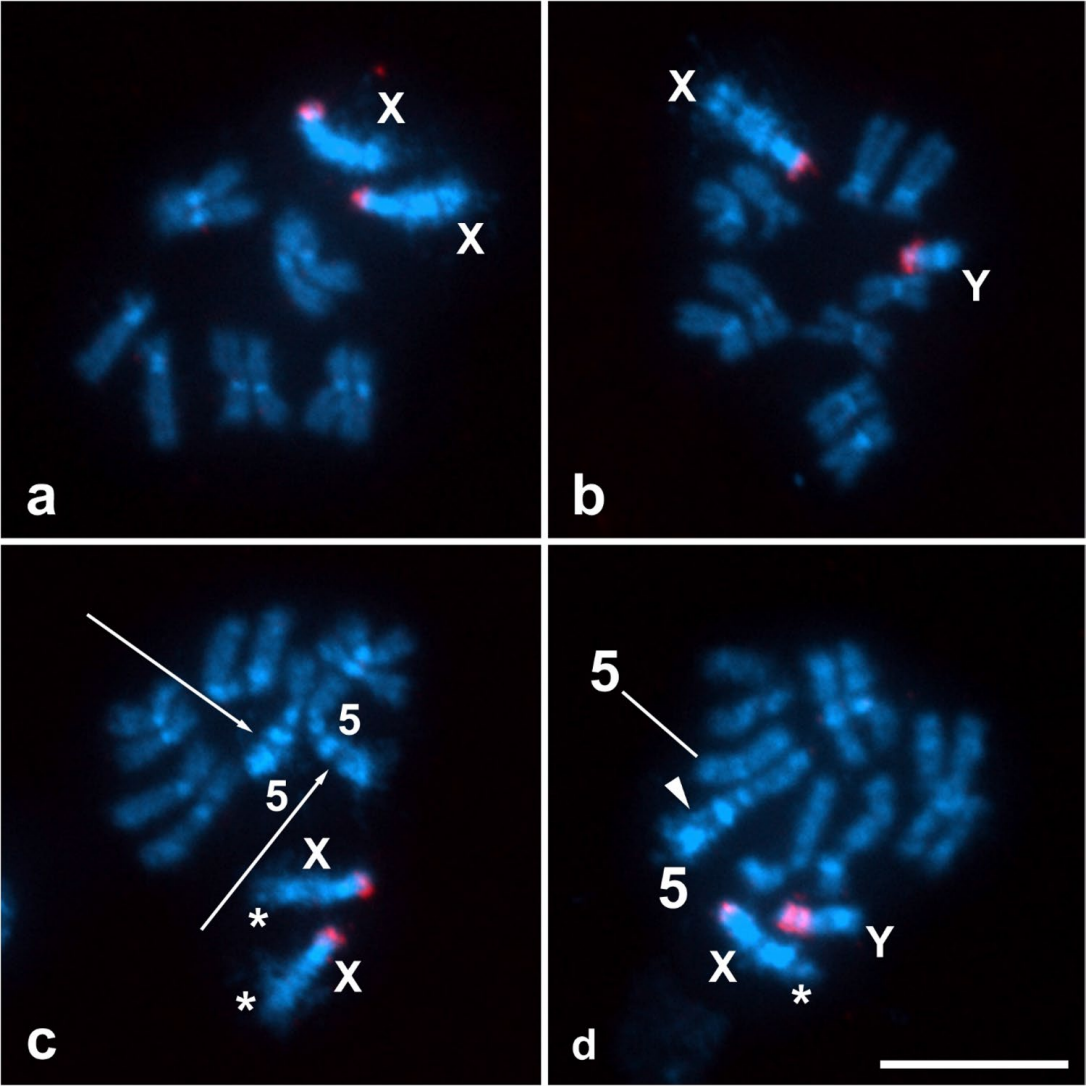
Localization of rDNA clusters in mitotic chromosomes of *Ceratitis* *capitata* by FISH with the 18 rDNA probe (red signals). Chromosomes, obtained from the brains of wild-type larvae (**a**, **b**) and T(X;5) larvae (**c**, **d**), were counterstained with DAPI (light blue). X and Y symbols denote sex chromosomes, symbol 5 stands for chromosome 5. Bar = 10 µm. (**a**) Female mitotic metaphase with two X chromosomes, each with a terminal rDNA cluster, and five pairs of autosomes. (**b**) Male mitotic metaphase with X and Y chromosomes, each with a terminal rDNA cluster, and five pairs of autosomes. (**c**) Female mitotic metaphase showing reciprocal translocations between two X chromosomes (asterisks), each with a terminal cluster of rDNA, and two chromosomes 5 (arrows). (**d**) Male mitotic prometaphase showing a T(X;5) translocation on the X chromosome (asterisk) and a T(5;X) translocation on one chromosome 5 (arrowhead); both the X and Y chromosomes show terminal clusters of rDNA (note that the rDNA clusters on the Y chromosome appear duplicated).

In preparations of mitotic chromosomes of both sexes of the *C.* *capitata* wild-type strain , FISH with the biotinylated 18S rDNA probe localized the major rDNA exclusively to the sex chromosomes (Fig. 5a,b) in agreement with previously published data^37^. In female metaphase complements, the rDNA site was detected in the terminal region of the short arm of both acrocentric X chromosomes, and twin hybridization signals of the probe were frequently observed, each representing the rDNA site of one sister chromatid (Fig. 5a). In male metaphase complements, in addition to the hybridization signals of the rDNA probe at the end of the X chromosome, the rDNA site was also localized in the terminal region of the short arm of the acrocentric Y chromosome (Fig. 5b).

Similar results of FISH mapping of the major rDNA were also obtained in both sexes of the *C.* *capitata* translocation strain (Fig. 5c,d). In female mitotic metaphases, both X chromosomes showed hybridization signals of the 18S rDNA probe at the end of the short arm and a T(X;5) translocation at the opposite end of the X chromosomes, i.e. in the terminal region of the long arm (Fig. 5c). No hybridization signals were observed on the reciprocal T(5;X) translocation in either homologue of chromosome 5 (see arrows in Fig. 5c). The X chromosome in male mitotic metaphases also showed the rDNA site at one end and the T(X;5) translocation at the other end. However, the Y chromosome showed very strong probe signals covering almost the entire short arm, and the signal clusters often appeared duplicated (Fig. 5d). Similar to the female metaphases, the T(5;X) translocation, which is heterozygous in males (see above), was devoid of any hybridization signals (see arrowhead in Fig. 5d). The fact that the X-linked major rDNA is not involved in reciprocal translocation may be important for maintaining the overall performance of the *C.* *capitata* translocation strain.

## Discussion

Genetic sexing strains have been developed for several tephritid fruit fly pest species by combining: (a) natural occurring or induced mutations in autosomal genetic loci such as the *wp*, *bp* and *tsl* as selectable markers and (b) irradiation-induced translocations T(Y;A), which allow the pseudo-linkage of the wild- type allele of the selectable markers with the male determining region^12–20,28^. GSS based on these approaches are fully functional and have been used to mass-produce, on a weekly basis, billions of sterile males for male-only releases and SIT applications against major agricultural pests since several decades^8,25,26^.

In this study, we developed an innovative GSS for *C.* *capitata* based on a homozygous reciprocal translocation between chromosomes X and 5 [T(X;5)], and using the same selectable markers, *wp* and *tsl*, as for the VIENNA GSS. One important condition for the establishment of this GSS is that the translocation must be viable in homozygosity, so the females can express the white pupae and temperature sensitive lethal phenotypes. Males heterozygous for the translocation [T(X;5)] have a free chromosome 5 carrying the dominant alleles of the selectable markers and express the wild-type phenotype.

Cytogenetic analysis of the Cc TX IPCL-3^D53-^ strain confirmed the structure of this new GSS. The females are homozygous for the reciprocal translocation between the terminal segments of the long arm of the acrocentric X chromosome and the short arm of the submentacentric chromosome 5. The segment of chromosome 5 translocated onto the X chromosome is relatively short, accounting for less than 20% of the length of the T(X;5) chromosome, whereas the translocation of the X chromosome onto chromosome 5 is much larger, accounting for about 40% of the length of the T(5;X) chromosome (Figs. 4c,d, 5c). As expected, GSS males are hemizygous for the same X chromosome carrying the T(X;5) translocation as females and heterozygous for the translocation of the X chromosome segment onto chromosome 5 (Figs. 4e,f, 5d). In contrast, the Y chromosome and one of the homologues of the chromosome 5 pair in males show no rearrangement and appear to have remained intact, i.e. wild-type. The structure of this strain was also confirmed by FISH with the 18S rDNA probe. Moreover, FISH mapping clearly showed that the major rDNA site, located at the ends of the short arms of both X and Y chromosomes, was not involved in reciprocal translocation (Fig. 5). Therefore, the cytogenetic analysis is coincident with the hypothetical structure of this new sexing system shown in Fig. 1. The major rDNA has also been found on sex chromosomes in other tephritid species^38–41^ and sex-linkage of genes encoding ribosomal RNA seems to be a general feature also for other dipteran groups^42,43^. The proper function of ribosomal genes is vital for all organisms, and as has been shown in *Drosophila* *melanogaster*, the sex-linked location of rDNA may play an important role in the achiasmatic pairing of X and Y chromosomes in male meiosis^44^.

Although the TX GSS described here required more time for their development, as the T(X;A) translocation has to be in homozygosity, they could eventually replace the T(Y;A)-based GSS because they have several advantages. First, males and females of the homozygous T (X;5) translocation develop faster than males and females of the VIENNA 8^D53-^ strain. Both the Cc TX IPCL-1^D53-^ and Cc TX IPCL-2^D53^ strains show a faster development pattern, which is comparable to that of the VIENNA 8^D53-^ FD (fast development) strain reported recently^45^. Third, it is important to note that exposing embryos to 35 °C induced 100% female lethality without a significant reduction in male recovery rate. Second, both TX strains showed higher pupal and adult recovery rates as compared with the VIENNA 8^D53-^ strain, irrespective of the heat treatment. This means that the recovery rates were better under both normal production (males and females) and male-only production. Fourth, the mating competitiveness of males of the T(Y;5) and T(X;5) GSS was equal or better than that of the wildish males from Guatemala or Spain when tested under field cage conditions. However, fecundity of both Cc TX strains was significantly reduced as compared with the VIENNA 8^D53-^ strain, perhaps due to an effect the T(X;A) translocation may have on female meiosis^46^. Both advantages and disadvantages need to be assessed under mass-rearing conditions, to better determine the overall productivity, biological quality, and cost-effectiveness of the TX strains^47,48^.

However, the T(X;A) GSS offers an additional advantage. It has been shown that colonization^49^ and mass-rearing under artificial holding conditions may reduce the biological quality of a strain including male mating competitiveness^32^. In view that the T(X;5) translocation is in homozygosity and the females carry the genes encoding the selectable markers, the genetic refreshment of the mass-reared strain will be greatly facilitated. GSS females can be crossed *en* *masse* with wild or wildish males collected from the target area. F_1_ males will carry the translocation as they will have inherited it from their mothers, and they can be directly backcrossed with GSS females to re-establish the T(X;5) GSS strain (or any GSS strain carrying a selectable marker in an autosome). The re-established GSS will now carry “fresh” genetic material from the wild. This crossing scheme can be established periodically to maintain a high competitiveness of the GSS flies^32^.

A major challenge for GSSs is genetic recombination. All *C.* *capitata* VIENNA GSS developed so far are based on T(Y;A) translocations, and three types of recombination have been reported^8,27^: (a) type 1a, which occurs between the translocation breakpoint and the *wp* locus; (b) type 1b, which takes place between the two selectable markers, *wp* and *tsl* and (c) type 2, which is the rarest one and occurs in areas of repetitive sequences of translocated and not translocated Y chromosomal fragments^40^. The first two types of recombination can be addressed using a filter colony for the removal of recombinants^30^. However, type 2 recombination will generate individuals with free Y and such males are fertile and can accumulate very fast. In addition, matings of these males with wild-type recombinant females will result in the eventual reversing of the sexing character of the strain (brown pupae females, white pupae males) and its genetic breakdown^8^. The new strains are stable under small-scale rearing conditions. However, their long-term genetic stability, and their overall performance, needs to be closely monitored and tested under large-scale rearing conditions prior to their use in operational programs.

The new GSS based on homozygous T(X;5) translocation may further optimize the mass-rearing process and cost-effectiveness of ongoing operational SIT programs and may also facilitate the implementation of new ones. This is because they show three key advantages in comparison with the currently used T(Y;5)-based VIENNA 8 GSS, i.e., (a) a higher pupal production rate, (b) a faster development time of the females and, (c) easier and faster refreshment of the mass-reared colony with insects of a local genetic background. The adoption of the new strains by the mass-rearing facilities will also require the establishment of a mother colony of a least a few hundred insects but ideally more than 1000 pairs, to facilitate the genetic refreshment steps before the insects move to the actual production process. It is also important to note that this new genetic sexing system can be applied to any insect species with an XY or similar sex determination system and at least one selectable marker.

## Methods

### Study site and strains

The experiments were carried out at the Insect Pest Control Laboratory (IPCL) of the Joint FAO/IAEA Centre of Nuclear Techniques in Food and Agriculture, Seibersdorf, Austria. For the development and evaluation of the novel GSS T(X;5), the following Mediterranean fruit fly strains were used: (a) the wild-type strain Egypt II; (b) the double mutant strain *wp* *tsl*, which carries the *white* *pupae* and *temperature* *sensitive* *lethal* genes. Insects homozygous for these markers have a white color puparium and their embryos die when exposed to temperatures of 34–35 °C^8^; (c) the inversion line D53 (namely D53 *wp* *tsl*) covers a large part of chromosome 5 (50B-59C based on trichogen polytene chromosome map), it is homozygous for the *wp* and *tsl* mutant alleles, and its right chromosomal break point is located between these two genetic loci^8,20^; (d) the VIENNA 8 ^D53-^ GSS from the El Pino mass-rearing facility in Guatemala, which was used as a reference strain for the evaluation of the biological characteristics of the newly developed Mediterranean fruit fly T(X;5) GSS; (e) a wildish strain from Valencia, Spain maintained in the IPCL for 10 generations, and (f) a wildish strain from Guatemala, maintained in the IPCL for 11 generations. The two wildish strains were used in the male mating competitiveness test. All Mediterranean fruit fly strains were maintained at the IPCL under standard laboratory rearing conditions as described previously^50^.

### Development of homozygous viable T (X;5) homozygous translocations with or without D53 inversion

Mature pupae of the homozygous *wp* *tsl* or the D53 *wp* *tsl* strains that were close to adult emergence were irradiated with 40 Gy using a ^60^Co source (Gamma-cell 220, Nordion, Canada) for the development of the new GSS without or with the inversion D53, respectively^12^. After emergence, irradiated *wp* *tsl* (or D53 *wp* *tsl*) males were crossed *en* *masse* with wild-type Egypt II females. F_1_ females were then backcrossed with wild-type Egypt II males thus establishing 250 single pair crosses. Twenty males and twenty females (siblings) from each of the 250 families were crossed *en* *masse*. Crosses which produced both brown pupae and white pupae were initially selected, and the pupae were separated by color. We only kept families whose white pupae were only producing females while brown pupae were producing either males or females. As only a proportion of males caried the [T(X;5)] translocation at this stage, an additional step was required to establish a GSS based on [T(X;5)] translocation using the *wp* *tsl* selectable markers: fifteen *wp* females [T(X;5)] were crossed individually with wild-type (*wp*^+^) sibling males [T(X;5)], and the progeny of these single pair crosses were then evaluated with respect to their sensitivity to elevated temperatures. Subfamilies with females emerging from white pupae and being sensitive (die) at elevated temperatures and males emerging from brown pupae and being resistant at elevated temperatures were selected as GSS with a T(X;5)-based sexing system for further evaluation regarding their genetic stability and biological characteristics.

### Fecundity

Twenty-five single pair crosses were set up with newly emerged, virgin males and females, each in a two-side fine mesh plastic cubic cage (0.25 L capacity) for the three GSS: VIENNA 8^D53-^, Cc TX IPCL-1^D53-^, Cc TX IPCL-2^D53^. Insects were provided with a mixture of yeast hydrolysate and sugar (1:3) and water through a moist sponge. The fine mesh of the cages was used as an oviposition panel and eggs were collected for the first 12 days of oviposition in a Petri dish (10 cm diameter) that contained water to simulate conditions in a mass-rearing facility^25,50^. The number of eggs produced per female per day was recorded.

### Egg hatch, egg to pupal and egg to adult production rates

One thousand eggs from each of the three GSS, T (Y;5) VIENNA 8 ^D53-^, Cc TX IPCL-1^D53-^ and Cc TX IPCL-2^D53^, were incubated for 48 h on a black nylon net placed on a moist sponge in a Petri dish. After incubation, mature eggs were transferred to 200 g of carrot diet in a 20 cm diameter Petri dish. Three days after incubation, egg hatch was determined, and 5 days after incubation, the Petri dish lid was removed, and the Petri dish was transferred to a plastic box with ventilated fine mesh net lid and with its bottom covered with sawdust. Mature larvae that left the diet use the sawdust as a pupation medium^45^. Mature larvae and pre-pupa individuals were collected daily, and after the completion of ecdysis, the number of brown and white pupae were determined. Brown and white pupae were daily collected and placed separately in Petri dishes for recording adult emergence and sex. This experiment was carried out at 23 ± 1 °C, 65% RH and was replicated seven times.

### Temperature sensitivity

Temperature sensitivity of the three GSS was assessed by exposing 1000 eggs from each strain to 24 °C or 35 °C. Each thermal treatment was replicated five times. For the 35 °C treatment, the eggs were first incubated at 24 °C for 24 h, then at 35 °C for 24 h and were then incubated at 24 °C to complete their development. The number of wild type (brown) and white pupae was recorded, and it was used as response variable to assess the temperature sensitivity^8,25^.

### Male mating competitiveness in walk-in field cages

Eight walk-in field cages made of nylon netting mesh (3 m diameter × 2 m height) supported by a frame of PVC pipes^51^ were set-up in an insect greenhouse with controlled environmental conditions of 25–26 °C, 45–60% R.H and light intensity range between 1000 and 2000 Lux. One potted orange tree (*Citrus* *sinensis*) was placed into each field cage^51^. The mating competitiveness of fertile males of the T (Y;5) VIENNA 8^D53-^, GSS Cc TX IPCL-1^D53-^, GSS Cc TX IPCL-2^D53^ GSS was compared against Valencia F10 and Guatemala F11 wildish males while competing for Valencia F10 or Guatemala F11 females, respectively. The male mating competitiveness tests were carried out with 5–6 day-old GSS males and 8 day-old wildish flies (males and females). The day before emergence, the male pupae from the three GSS were marked with fluorescent dye powder^48^. The field cages used for the experiment were rotated between the three GSS to eliminate any bias. On the experimental days, 50 males (25 GSS and 25 wildish) and 25 females were released into each of the respective GSS field cages, at 07:30 and 08:30 h respectively. The field cages were inspected every 20 min until 14:00 h. Every couple detected was placed in a transparent glass vial where it was kept for subsequent identification under a fluorescent stereomicroscope to determine the type of the male (GSS or wildish). The experiment was replicated eight times for each GSS.

The citrus trees (*Citrus* *sinensis*) mentioned above, were purchased from a local market in Vienna. These trees are maintained in our greenhouse and are only used in quality assessment studies as natural mating arenas of *Ceratitis* *capitata*. The trees have not been subject of any experimental treatment; they were not imported; and they are not in the list of threatened species. Consequently, no specific permission was required to use these plants.

### Chromosome preparation

Spread preparations of mitotic chromosomes from the wild-type strain and the Cc TX IPCL-3^D53-^ GSS were made from the brain (cerebral ganglia) of third instar larvae using a previously described method^41,52^ with slight modifications. Briefly, the brain was dissected in a physiological solution, transferred into hypotonic solution (0.075 M KCl) for 10 min, and then fixed in freshly prepared Carnoy’s fixative (ethanol, chloroform, acetic acid; 6:3:1) for 15 min. The fixed tissue was spread on the slide in a drop of 60% acetic acid at 45 °C using a heating plate. Then the preparations were passed through a graded ethanol series (70%, 80%, and 100%, 30 s each), air dried, and stored at − 20 °C. Before further use, the preparations were dehydrated again in the ethanol series immediately after removal from the freezer.

The preparations were stained and mounted in antifade based on DABCO (1,4‐diazabicyclo (2.2.2)‐octane)^52^, containing 0.5 μg/ml DAPI (4′,6‐diamidino‐2‐phenylindole) (both Sigma‐Aldrich, St. Louis, MO, USA), and observed in a Zeiss Axioplan 2 microscope (Carl Zeiss, Jena, Germany). Digital images were captured with an Olympus CCD monochrome camera XM10 equipped with cellSens 1.9 digital imaging software (Olympus Europa Holding, Hamburg, Germany) and processed with Adobe Photoshop CS4 (Adobe Systems Inc., San Jose, CA, USA).

### Fluorescence in situ hybridization (FISH) with 18S rDNA probe

Unlabelled 18S ribosomal DNA (rDNA) probe was prepared by PCR with universal arthropod primers, forward 5′-CCTGAGAAACGGCTACCACATC-3´ and reverse 5′-GAGTCTCGTTCGTTATCGGA-3′^53^, custom-made by Generi Biotech (Hradec Králové, Czech Republic). Total genomic DNA (gDNA) of *C.* *capitata*, obtained by standard phenol–chloroform–isoamyl alcohol extraction, served as template.

PCR was performed essentially according to the procedure described in a previous study^54^ with some modifications. The PCR product showed a single band of about 1000 bp. The band was purified using an ExoSAP-IT PCR Product Cleanup Reagent (Thermo Fisher Scientific, Waltham, MA, USA) and used as a template for PCR amplification of the 18S rDNA probe. The amplified product was verified by Sanger sequencing (SEQme, Dobříš, Czech Republic). Then the probe was labelled by PCR with biotin-16-dUTP (Jena Bioscience, Jena, Germany).

FISH with a biotinylated 18S rDNA probe was performed essentially following the universal protocol for mapping repetitive DNAs in insects^55^ with slight modifications. Briefly, denaturation of chromosomes was performed at 68 °C for 3.5 min in 70% deionized formamide in 2 × SSC. The probe cocktail for one slide (10 μl; 50% deionized formamide, 10% dextran sulfate in 2 × SSC) contained 50–100 ng of labelled probe and 25 μg of sonicated salmon sperm DNA. Hybridization was performed overnight at 37 °C. Hybridization signals were detected with Cy3-conjugated streptavidin (Jackson ImmunoRes. Labs. Inc., West Grove, PA, USA). Preparations were counterstained with 0.5 μg/ml DAPI in DABCO-based antifade and observed in a Zeiss Axioplan 2 microscope equipped with appropriate filter sets. Digital images were captured and processed as described above.

### Statistical analyses

Data were analyzed with R software (version 4.1 0.1)^56^. Negative binomial general linear model was used to analyses the egg production per female with the number of eggs per female as response variable, the GSS as fixed effect and the day of egg collection and the replicates as random effects. The time to pupation was analysed using Gaussian linear mixed-effects model with the larval development time from egg hatch to pupation assigned as response variable, the GSS and sex as fixed effects. The replicates were considered as random effects. The egg hatch rate and the production of pupae and adults were analyzed using binomial generalized linear mixed models fit by maximum likelihood (Laplace Approximation)^57^. Egg hatching, pupal and adult recovery were considered as response variables, the GSS and the temperature as fixed effects and the replicates as random effect. When the best fit linear mixed model was selected^58^ and a significant effect was detected, pairwise comparisons were performed between the levels of the factors using the “emmeans” package.

The mating proportion (PM = number of mating pairs collected/number of females released) was estimated for the two wild-type population, because for SIT field applications, it is important to have a PM > 0.2^59^. On another hand, a one-way analysis (pooled *t*-test and ANOVA) was performed with the number of mating recorded from six replicates performed, to determine significant differences in mating between GSS and wildish male for each GGS strain, using the two wild populations. In addition, the relative sterility index (RSI = number of matings achieved by males of a given GSS/total number of matings achieved by wildish females) was used to estimate the sexual competitiveness of each GSS. The RSI values range between 0 and 1 and values close to 0.50 indicate equal sexual performance between GSS and wildish males^60^. The RSI values of each GSS were assessed using an analysis of variance (ANOVA) at 5% significant level, for the Guatemala and Valencia wild type population. The field cage data were analysed using the JMP® Pro 17.0.0 software^61^.

### Ethics statements

Experimental research and field studies on plants (either cultivated or wild), including the collection of plant material were carried out in accordance with relevant institutional, national, and international guidelines and legislation.

### Supplementary Information

Supplementary Information 1.

Supplementary Information 2.

## Data Availability

The datasets used and/or analysed during the current study available from the corresponding author on reasonable request.
