# Supplementary material for: Development of a novel genetic sexing strain of Ceratitis capitata based on an X-autosome translocation
Source: Sci Rep. 2023 Sep 27;13:16167. doi: 10.1038/s41598-023-43164-0 (PMC10533888; doi:10.1038/s41598-023-43164-0)
Supplement: Supplementary file 2 — Supplementary Information 2. [file 41598_2023_43164_MOESM2_ESM.docx]

**Statistics supplementary information by each respective experiment**

**Fecundity - Egg production per female**

Mean number of eggs per female per day

| **Strain** | **Mean** | **sem** |
| --- | --- | --- |
| **VIENNA 8^D53-^** | 41.06 | 1.79 |
| **Cc TX -1^D53-^** | 34.48 | 1.59 |
| **Cc TX -2^D53^** | 35.71 | 1.44 |

|  | **Chisq** | **Df** | **Pr(>Chisq)** |
| --- | --- | --- | --- |
| **Strain** | 166.63 | 2 | < 2.2E-16 |

Pairwise comparison of the mean number of eggs per female per day

| **contrast** | **ratio** | **SE** | **df** | **null** | **z.ratio** | **p.value** |
| --- | --- | --- | --- | --- | --- | --- |
| **VIENNA 8^D53-^ / Cc TX -1^D53-^** | 1.191 | 0.0173 | Inf | 1 | 12.024 | <.0001 |
| **VIENNA 8^D53-^ / Cc TX -2^D53^** | 1.15 | 0.0165 | Inf | 1 | 9.709 | <.0001 |
| **Cc TX -1^D53-^ / CcTX -2^D53^** | 0.966 | 0.0145 | Inf | 1 | -2.327 | 0.0521 |

**Egg hatch rate**

Mean number of egg hatch rate

| **Strain** | **Mean** | **sem** |
| --- | --- | --- |
| **VIENNA 8^D53-^** | 87.61 | 0.55 |
| **Cc TX -1^D53-^** | 91.21 | 1.38 |
| **Cc TX -2^D53^** | 92.01 | 0.94 |

|  | **Chisq** | **Df** | **Pr(>Chisq)** |
| --- | --- | --- | --- |
| **Strain** | 86.793 | 2 | < 2.2e-16 *** |

Pairwise comparison of egg hatch rate

| **contrast** | **odds.ratio** | **SE** | **df** | **null** | **z.ratio** | **p.value** |
| --- | --- | --- | --- | --- | --- | --- |
| **VIENNA 8^D53-^ / Cc TX -1^D53-^** | 0.681 | 0.0379 | Inf | 1 | -6.901 | <.0001 |
| **VIENNA 8^D53-^ / Cc TX -2^D53^** | 0.613 | 0.035 | Inf | 1 | -8.555 | <.0001 |
| **Cc TX -1^D53-^ / CcTX -2^D53^** | 0.901 | 0.055 | Inf | 1 | -1.709 | 0.2018 |

**Larva development time**

**Mean larval development time (day) to pupation**

| **Strain** | **Pupae colour** | **Mean time** | **sem** |
| --- | --- | --- | --- |
| **VIENNA 8^D53^** | Brown | 10.09 | 0.05 |
| **VIENNA 8^D53^** | White | 13.23 | 0.05 |
| **Cc TX -1^D53-^** | Brown | 8.81 | 0.21 |
| **Cc TX -1^D53-^** | White | 9.71 | 0.18 |
| **Cc TX -2^D53^** | Brown | 8.35 | 0.06 |
| **Cc TX -2^D53^** | White | 9.10 | 0.08 |

Analysis of Deviance Table (Type II tests)

Response: Time

Chisq Df Pr(>Chisq)

Strain 901.64 2 < 2.2e-16 ***

Colour 353.26 1 < 2.2e-16 ***

Strain:Colour 165.48 2 < 2.2e-16 ***

**Larva development time: Comparison of strains within each pupa colour/sex**

|  | **contrast** | **estimate** | **SE** | **df** | **t.ratio** | **p.value** |
| --- | --- | --- | --- | --- | --- | --- |
| **Brown pupae -males** | **VIENNA 8^D53-^ / Cc TX -1^D53-^** | 1.278 | 0.159 | 30 | 8.033 | <.0001 |
|  | **VIENNA 8^D53-^ / Cc TX -2^D53^** | 1.741 | 0.159 | 30 | 10.945 | <.0001 |
|  | **Cc TX -1^D53-^ / Cc TX -2^D53^** | 0.463 | 0.159 | 30 | 2.912 | 0.0179 |
|  |  |  |  |  |  |  |
| **White pupae - females** | **VIENNA 8^D53-^ / Cc TX -1^D53-^** | 3.520 | 0.159 | 30 | 22.132 | <.0001 |
|  | **VIENNA 8^D53-^ / Cc TX -2^D53^** | 4.131 | 0.159 | 30 | 25.974 | <.0001 |
|  | **Cc TX -1^D53-^ / Cc TX -2^D53^** | 0.611 | 0.159 | 30 | 3.842 | 0.0017 |

**Larva development time: Comparison of pupa colours within each strain**

| **Strain** | **contrast** | **estimate** | **SE** | **df** | **t.ratio** | **p.value** |
| --- | --- | --- | --- | --- | --- | --- |
| **VIENNA 8^D53-^** | Brown - White | -3.142 | 0.159 | 30 | -19.756 | <.0001 |
| **Cc TX -1^D53-^** | Brown - White | -0.900 | 0.159 | 30 | -5.657 | <.0001 |
| **Cc TX -2^D53^** | Brown - White | -0.752 | 0.159 | 30 | -4.727 | 0.0001 |

**Experiment 2 : Total pupae production from eggs**

Chisq Df Pr(>Chisq)

Strain 555.79 2 < 2.2e-16 ***

| **Contrast** | **odds.ratio** | **SE** | **df** | **null** | **z.ratio** | **p.value** |
| --- | --- | --- | --- | --- | --- | --- |
| **VIENNA 8^D53-^ / Cc TX -1^D53-^** | 0.427 | 0.0196 | Inf | 1 | -18.532 | <.0001 |
| **VIENNA 8^D53-^ / Cc TX -2^D53^** | 0.385 | 0.0181 | Inf | 1 | -20.252 | <.0001 |
| **Cc TX -1^D53-^ / Cc TX -2^D53^** | 0.902 | 0.0475 | Inf | 1 | -1.948 | 0.1255 |

**Egg to pupae production rate (%)**

**(Males + females)**

Chisq Df Pr(>Chisq)

Strain 436.49 2 < 2.2e-16 ***

Pairwise comparison of adult production (total Males + females)

| **contrast** | **odds.ratio** | **SE** | **df** | **null** | **z.ratio** | **p.value** |
| --- | --- | --- | --- | --- | --- | --- |
| **VIENNA 8^D53-^ / Cc TX -1^D53-^** | 0.467 | 0.0191 | Inf | 1 | -18.608 | <.0001 |
| **VIENNA 8^D53-^ / Cc TX -2^D53^** | 0.520 | 0.0209 | Inf | 1 | -16.286 | <.0001 |
| **Cc TX -1^D53-^ / Cc TX -2^D53^** | 1.115 | 0.0491 | Inf | 1 | 2.464 | 0.0366 |

**Experiment 2 : Comparison adult production by strain by sex**

|  | SumSq | Df | F value | Pr(>F) |
| --- | --- | --- | --- | --- |
| **Strain** | 58.402 | 2 | 25.6389 | 1.194e-07 *** |
| **Sex** | 27.556 | 1 | 24.1948 | 1.925e-05 *** |
| **Strain:Sex** | 8.707 | 2 | 3.8223 | 0.03124* |
| **Residuals** | 41.001 | 36 |  |  |

**Experiment 2 : Comparison adult production by strain within each sex**

|  | **contrast** | **ratio** | **SE** | **df** | **null** | **z.ratio** | **p.value** |
| --- | --- | --- | --- | --- | --- | --- | --- |
| **Female** | **VIENNA 8^D53-^ / Cc TX -1^D53-^** | 0.797 | 0.0297 | Inf | 1 | -6.087 | <.0001 |
|  | **VIENNA 8^D53-^ / Cc TX -2^D53^** | 0.787 | 0.0292 | Inf | 1 | -6.458 | <.0001 |
|  | **Cc TX -1^D53-^ / Cc TX -2^D53^** | 0.987 | 0.0354 | Inf | 1 | -0.373 | 0.9263 |
|  |  |  |  |  |  |  |  |
| **Male** | **VIENNA 8^D53-^ / Cc TX -1^D53-^** | 0.871 | 0.0310 | Inf | 1 | -3.874 | 0.0003 |
|  | **VIENNA 8^D53-^ / Cc TX -2^D53^** | 0.916 | 0.0329 | Inf | 1 | -2.452 | 0.0378 |
|  | **Cc TX -1^D53-^ / Cc TX -2^D53^** | 1.051 | 0.0370 | Inf | 1 | 1.425 | 0.3282 |

**Experiment 2 : Comparison adult production by sex within each strain**

|  | **contrast** | **ratio** | **SE** | **df** | **null** | **z.ratio** | **p.value** |
| --- | --- | --- | --- | --- | --- | --- | --- |
| **VIENNA 8^D53-^** | Female / Male | 0.825 | 0.0309 | Inf | 1 | -5.138 | <.0001 |
| **Cc TX -1^D53-^** | Female / Male | 0.902 | 0.0320 | Inf | 1 | -2.923 | 0.0035 |
| **Cc TX -2^D53^** | Female / Male | 0.961 | 0.0342 | Inf | 1 | -1.126 | 0.2600 |

**Temperature sensitivity**

**Means after treatment at 35 degres**

|  | **Mean egg hatch rate** | **se** | **Mean brown pupae rate** | **se** | **Mean adult male production rate** | **se** |
| --- | --- | --- | --- | --- | --- | --- |
| **VIENNA 8^D53-^** | 50.44 | 1.10 | 37.07 | 1.03 | 34.69 | 1.04 |
| **Cc TX -1^D53-^** | 46.40 | 0.70 | 41.09 | 1.22 | 38.40 | 0.93 |
| **Cc TX -2^D53^** | 46.43 | 1.40 | 43.07 | 1.80 | 40.21 | 1.60 |

**Egg hatch rate** **35 degrees**

| **contrast** | **odds.ratio** | **SE** | **df** | **null** | **z.ratio** | **p.value** |
| --- | --- | --- | --- | --- | --- | --- |
| **VIENNA 8^D53-^ / Cc TX -1^D53-^** | 1.176 | 0.0398 | Inf | 1 | 4.785 | <.0001 |
| **VIENNA 8^D53-^ / Cc TX -2^D53^** | 1.175 | 0.0398 | Inf | 1 | 4.751 | <.0001 |
| **Cc TX -1^D53-^ / Cc TX -2^D53^** | 0.999 | 0.0339 | Inf | 1 | -0.034 | 0.9994 |

**Pupation rate from eggs 35 degrees**

Chisq Df Pr(>Chisq)

Strain 30.343 2 2.577e-07 ***

| **contrast** | **odds.ratio** | **SE** | **df** | **null** | **z.ratio** | **p.value** |
| --- | --- | --- | --- | --- | --- | --- |
| **VIENNA 8^D53-^ / Cc TX -1^D53-^** | 0.844 | 0.0293 | Inf | 1 | -4.870 | <.0001 |
| **VIENNA 8^D53-^ / Cc TX -2^D53^** | 0.778 | 0.0269 | Inf | 1 | -7.245 | <.0001 |
| **Cc TX -1^D53-^ / Cc TX -2^D53^** | 0.922 | 0.0316 | Inf | 1 | -2.382 | 0.0454 |

**Adult production from eggs 35 degrees**

Chisq Df Pr(>Chisq)

Strain 54.337 2 1.588e-12 ***

| **contrast** | **odds.ratio** | **SE** | **df** | **null** | **z.ratio** | **p.value** |
| --- | --- | --- | --- | --- | --- | --- |
| **VIENNA 8^D53-^ / Cc TX -1^D53-^** | 0.852 | 0.0299 | Inf | 1 | -4.564 | <.0001 |
| **VIENNA 8^D53-^ / Cc TX -2^D53^** | 0.789 | 0.0276 | Inf | 1 | -6.757 | <.0001 |
| **Cc TX -1^D53-^ / Cc TX -2^D53^** | 0.927 | 0.0321 | Inf | 1 | -2.199 | 0.0714 |

**Comparison between temperature treatments**

Comparison of male pupae production between temperature treatments

|  | Chisq | Df | Pr(>Chisq) |
| --- | --- | --- | --- |
| Temperature | 48.7417 | 1 | 2.92e-12 *** |
| Strain | 68.9415 | 2 | 1.07e-15*** |
| Temperature:Strain | 7.4735 | 2 | 0.02383* |

| **Strain** | **contrast** | **odds.ratio** | **SE** | **df** | **null** | **z.ratio** | **p.value** |
| --- | --- | --- | --- | --- | --- | --- | --- |
| **VIENNA 8^D53-^** | 24 / 35 | 0.830 | 0.0288 | Inf | 1 | -5.381 | <.0001 |
| **Cc TX -1^D53-^** | 24 / 35 | 0.846 | 0.0289 | Inf | 1 | -4.880 | <.0001 |
| **Cc TX -2^D53^** | 24 / 35 | 0.939 | 0.0320 | Inf | 1 | -1.857 | 0.0633 |

Comparison of male adults’ production between temperature treatments

|  | Chisq | Df | Pr(>Chisq) |
| --- | --- | --- | --- |
| Temperature | 44.558 | 1 | 2.47e-11*** |
| Strain | 82.435 | 2 | <2.2e-16*** |
| Temperature:Strain | 11.296 | 2 | 0.003525*** |

| **Strain** | **contrast** | **odds.ratio** | **SE** | **df** | **null** | **z.ratio** | **p.value** |
| --- | --- | --- | --- | --- | --- | --- | --- |
| **VIENNA 8^D53-^** | 24 / 35 | 0.870 | 0.0306 | Inf | 1 | -3.972 | 0.0001 |
| **Cc TX -1^D53-^** | 24 / 35 | 0.809 | 0.0278 | Inf | 1 | -6.168 | <.0001 |
| **Cc TX -2^D53^** | 24 / 35 | 0.952 | 0.0328 | Inf | 1 | -1.427 | 0.1534 |

| **Male mating competitivenessComparison number of mating Guatemala *vs* Cc TX -1^D53-^** | | | | | | | | | | |
| --- | --- | --- | --- | --- | --- | --- | --- | --- | --- | --- |
|  |  |  | |  | |  | |  | |  |
| Pooled t Test (Assuming equal variances). | | | | | | |  | |  | |
| Difference | -3.8333 | t Ratio | | -5.45152 | |  | |  | |  |
| Std Err Dif | 0.7032 | DF | | 10 | |  | |  | |  |
| Upper CL Dif | -2.2666 | Prob > \|t\| | | 0.0003* | |  | |  | |  |
| Lower CL Dif | -5.4001 | Prob > t | | 0.9999 | |  | |  | |  |
| Confidence | 0.95 | Prob < t | | 0.0001* | |  | |  | |  |
|  |  |  | |  | |  | |  | |  |
| Analysis of Variance | | |  | |  | |  | |  | |
| **Source** | **DF** | **Sum of Squares** | | **Mean Square** | | **F Ratio** | | **Prob > F** | |  |
| Strain | 1 | 44.083333 | | 44.0833 | | 29.7191 | | 0.0003* | |  |
| Error | 10 | 14.833333 | | 1.4833 | |  | |  | |  |
| C. Total | 11 | 58.916667 | |  | |  | |  | |  |
|  |  |  | |  | |  | |  | |  |
|  |  |  | |  | |  | |  | |  |
| **Comparison number of mating Guatemala *vs* Cc TX -2^D53^** | | | | | | | | | | |
|  |  |  | |  | |  | |  | |  |
| Pooled t Test (Assuming equal variances). | | | | | | |  | |  | |
| Difference | -3.6667 | t Ratio | | -5.5 | |  | |  | |  |
| Std Err Dif | 0.6667 | DF | | 10 | |  | |  | |  |
| Upper CL Dif | -2.1812 | Prob > \|t\| | | 0.0003* | |  | |  | |  |
| Lower CL Dif | -5.1521 | Prob > t | | 0.9999 | |  | |  | |  |
| Confidence | 0.95 | Prob < t | | 0.0001* | |  | |  | |  |
|  |  |  | |  | |  | |  | |  |
| Analysis of Variance | | |  | |  | |  | |  | |
| **Source** | **DF** | **Sum of Squares** | | **Mean Square** | | **F Ratio** | | **Prob > F** | |  |
| Strain | 1 | 40.333333 | | 40.3333 | | 30.25 | | 0.0003* | |  |
| Error | 10 | 13.333333 | | 1.3333 | |  | |  | |  |
| C. Total | 11 | 53.666667 | |  | |  | |  | |  |
|  |  |  | |  | |  | |  | |  |
|  |  |  | |  | |  | |  | |  |
| **Comparison number of mating Guatemala *vs* VIENNA 8^D53-^** | | | | | | | | | | |
|  |  |  | |  | |  | |  | |  |
| Pooled t Test (Assuming equal variances). | | | | | | |  | |  | |
| Difference | 3.5 | t Ratio | | 3.41565 | |  | |  | |  |
| Std Err Dif | 1.0247 | DF | | 10 | |  | |  | |  |
| Upper CL Dif | 5.78316 | Prob > \|t\| | | 0.0066* | |  | |  | |  |
| Lower CL Dif | 1.21684 | Prob > t | | 0.0033* | |  | |  | |  |
| Confidence | 0.95 | Prob < t | | 0.9967 | |  | |  | |  |
|  |  |  | |  | |  | |  | |  |
| Analysis of Variance | | |  | |  | |  | |  | |
| **Source** | **DF** | **Sum of Squares** | | **Mean Square** | | **F Ratio** | | **Prob > F** | |  |
| Strain | 1 | 36.75 | | 36.75 | | 11.6667 | | 0.0066* | |  |
| Error | 10 | 31.5 | | 3.15 | |  | |  | |  |
| C. Total | 11 | 68.25 | |  | |  | |  | |  |
|  |  |  | |  | |  | |  | |  |
| **Comparison number of mating_Valencia *vs* Cc TX -1^D53-^** | | | | | | | | |  | |
|  |  |  | |  | |  | |  | |  |
| Pooled t Test (Assuming equal variances). | | | | | | |  | |  | |
| Difference | -1.5 | t Ratio | | -1.60612 | |  | |  | |  |
| Std Err Dif | 0.9339 | DF | | 10 | |  | |  | |  |
| Upper CL Dif | 0.5809 | Prob > \|t\| | | 0.1393 | |  | |  | |  |
| Lower CL Dif | -3.5809 | Prob > t | | 0.9303 | |  | |  | |  |
| Confidence | 0.95 | Prob < t | | 0.0697 | |  | |  | |  |
|  |  |  | |  | |  | |  | |  |
| Analysis of Variance | | |  | |  | |  | |  | |
| **Source** | **DF** | **Sum of Squares** | | **Mean Square** | | **F Ratio** | | **Prob > F** | |  |
| Strain | 1 | 6.75 | | 6.75 | | 2.5796 | | 0.1393 | |  |
| Error | 10 | 26.166667 | | 2.61667 | |  | |  | |  |
| C. Total | 11 | 32.916667 | |  | |  | |  | |  |
|  |  |  | |  | |  | |  | |  |
|  |  |  | |  | |  | |  | |  |
| **Comparison number of mating_ Valencia *vs* Cc TX ^-D53^** | | | | | | | | |  | |
|  |  |  | |  | |  | |  | |  |
| Pooled t Test (Assuming equal variances). | | | | | | |  | |  | |
| Difference | 0.5 | t Ratio | | 0.361158 | |  | |  | |  |
| Std Err Dif | 1.3844 | DF | | 10 | |  | |  | |  |
| Upper CL Dif | 3.5847 | Prob > \|t\| | | 0.7255 | |  | |  | |  |
| Lower CL Dif | -2.5847 | Prob > t | | 0.3627 | |  | |  | |  |
| Confidence | 0.95 | Prob < t | | 0.6373 | |  | |  | |  |
|  |  |  | |  | |  | |  | |  |
| Analysis of Variance | | |  | |  | |  | |  | |
| **Source** | **DF** | **Sum of Squares** | | **Mean Square** | | **F Ratio** | | **Prob > F** | |  |
| Strain | 1 | 0.75 | | 0.75 | | 0.1304 | | 0.7255 | |  |
| Error | 10 | 57.5 | | 5.75 | |  | |  | |  |
| C. Total | 11 | 58.25 | |  | |  | |  | |  |
|  |  |  | |  | |  | |  | |  |
|  |  |  | |  | |  | |  | |  |
| **Comparison number of mating_ VIENNA 8D53- *vs* Valencia** | | | | | | | | |  | |
|  |  |  | |  | |  | |  | |  |
| Pooled t Test (Assuming equal variances). | | | | | | |  | |  | |
| Difference | 0.6667 | t Ratio | | 0.472719 | |  | |  | |  |
| Std Err Dif | 1.4103 | DF | | 10 | |  | |  | |  |
| Upper CL Dif | 3.809 | Prob > \|t\| | | 0.6466 | |  | |  | |  |
| Lower CL Dif | -2.4756 | Prob > t | | 0.3233 | |  | |  | |  |
| Confidence | 0.95 | Prob < t | | 0.6767 | |  | |  | |  |
|  |  |  | |  | |  | |  | |  |
| Analysis of Variance | | |  | |  | |  | |  | |
| **Source** | **DF** | **Sum of Squares** | | **Mean Square** | | **F Ratio** | | **Prob > F** | |  |
| Strain | 1 | 1.333333 | | 1.33333 | | 0.2235 | | 0.6466 | |  |
| Error | 10 | 59.666667 | | 5.96667 | |  | |  | |  |
| C. Total | 11 | 61 | |  | |  | |  | |  |
|  |  |  | |  | |  | |  | |  |
|  |  |  | |  | |  | |  | |  |
| **Analysis of Variance of RSI with Guatemala Population** | | | | | | | | |  | |
|  |  |  | |  | |  | |  | |  |
| **Source** | **DF** | **Sum of Squares** | | **Mean Square** | | **F Ratio** | | **Prob > F** | |  |
| Strains | 2 | 0.00007778 | | 0.000039 | | 0.0132 | | 0.9869 | |  |
| Error | 15 | 0.04428333 | | 0.002952 | |  | |  | |  |
| C. Total | 17 | 0.04436111 | |  | |  | |  | |  |
|  |  |  | |  | |  | |  | |  |
|  |  |  | |  | |  | |  | |  |
| **Analysis of Variance of RSI with Valencia Population** | | | | | | | | |  | |
|  |  |  | |  | |  | |  | |  |
| **Source** | **DF** | **Sum of Squares** | | **Mean Square** | | **F Ratio** | | **Prob > F** | |  |
| Strains | 2 | 0.0061 | | 0.00305 | | 0.4758 | | 0.6304 | |  |
| Error | 15 | 0.09615 | | 0.00641 | |  | |  | |  |
| C. Total | 17 | 0.10225 | |  | |  | |  | |  |
|  |  |  | |  | |  | |  | |  |
